# Assessment of Therapeutic Bio-Activity of Cinnamoyl Sulfonamide Hydroxamate in Squamous Cell Carcinoma

**DOI:** 10.7759/cureus.43949

**Published:** 2023-08-22

**Authors:** Eapen Cherian, Manoj Goyal, Neeti Mittal, Venu Yesodharan, Ramya Ramadoss, Cinu Thomas

**Affiliations:** 1 Oral Pathology and Oral Biology, Travancore Dental College, Kollam, IND; 2 Oral & Maxillofacial Surgery, Santosh Deemed to be University, Ghaziabad, IND; 3 Pediatric Dentistry, Santosh Deemed to be University, Ghaziabad, IND; 4 Oral and Maxillofacial Surgery, Travancore Dental College, Kollam, IND; 5 Oral Biology, Saveetha Dental College, Chennai, IND; 6 Pharmacy, Caritas College of Pharmacy, Kottayam, IND

**Keywords:** bio activity, anti-cancer, mtt assay, oral squamous cell carcinoma, cinnamoyl sulfonamide, cinnamon, cancer

## Abstract

Background

Cancer is the second most common cause of death. Oral squamous cell carcinoma (OSCC) represents the most frequent of all oral neoplasms. Many treatment modalities such as chemotherapy, radiotherapy, surgery, and immunotherapy are emerging but still, the patients' quality of life is questionable. Despite the advances in therapeutic approaches, the percentages of morbidity and mortality of OSCC have not improved significantly during the last 30 years. Treatment using natural products can act as a potent anti-cancer agent with reduced adverse effects. Cinnamic acid derivatives exhibit anti-cancer potential through histone deacetylase inhibitor (HDAC) enzyme inhibition.

Methodology

In an experimental study design, cinnamoyl hydroxamate derivatives were prepared. The structure was confirmed using ultraviolet-visible spectroscopy (UV-Vis), nuclear magnetic resonance (NMR), infrared spectroscopy, and mass spectrophotometry. An in-vitro antioxidant assay using nitric oxide scavenging and reducing power assay was done and an in-vitro cytotoxic (3-[4,5-dimethylthiazol-2-yl]-2,5 diphenyl tetrazolium bromide) (MTT) assay and viability assay were carried out using tryphan blue dye.

Results

Statistical analysis was performed using SPSS (IBM Corp. Released 2013. IBM SPSS Statistics for Windows, Version 22.0. Armonk, NY: IBM Corp). Cinnamoyl hydroxamate derivatives were obtained and named as compounds 3a (E)-N-Hydroxy-3-(4-(N-(phenyl bromo) sulfamoyl) phenyl) acrylamide-) and 3b ((E)-N-Hydroxy-3-(4-(N-(phenyl nitro) sulfamoyl) phenyl) acrylamide). In the nitric oxide scavenging assay, compound 3a showed good antioxidant activity than 3b. Reducing power assay was higher in 3a compared to 3b. Cell viability using tryphan blue exhibited a concentration decrease in % cell viability with an increase in the concentration of human oral cavity squamous cell carcinoma cell line (OECM 1), a unique head and neck squamous carcinoma cell line (UM SCC 6) & human oral squamous cell carcinoma forming metastatic foci (HSC 3) cell lines.

Conclusion

The results of the present study revealed that the study compounds play a vital role in the up-regulation of apoptotic pathways and regulation of terminal differentiation pathways. The compounds showed good anti-oxidant and anti-cancer activities in lesser concentrations, hence they can be used as a therapeutic agent for oral squamous cell carcinoma.

## Introduction

The major global cause of adult mortality is cancer. The term "oral cancer" refers to a diverse range of cancers that can develop in the mouth in various locations. More than 4.5 million people worldwide pass away from cancer each year, with an estimated nine million new cases being diagnosed each year. Oral cancer is one of the top three types of cancer in India, accounting for the second and third most prevalent types of cancer in men and women, respectively [1].

Uncertainty, exposure to harsh climatic conditions, and behavioral risk factors are signs of a broad range of incidences over the world. In addition to being a significant risk factor for oral cancer, periodontal diseases are more common in the Indian population, where the practice of chewing paan is a major contributing factor. Inflammation, which is brought on by bacterial and viral infections as well as inflammation, is involved in the development of tumors [1].

Oral squamous cell carcinogenesis is a multi-step process in which several genetic occurrences modify the oncogenes' and tumor suppressor genes' typical roles, disrupting the pathways normally used for regulatory control. This may lead to a rise in the production of transcription factors, growth factors, or the number of cell surface receptors, as well as improved intracellular messenger signaling. This causes a cell phenotype capable of enhanced cell proliferation, with a lack of cell cohesion, and the ability to infiltrate local tissue and spread to distant areas when combined with the loss of tumor suppressor activity [2].

Conventional diagnostic techniques like clinical and histopathological examination, vital staining, biopsy, and spectroscopic analysis are been used to detect oral cancer. But diagnosing cancer at an early stage will help in better survival and quality of life of the patients.

Based on the staging of the cancer, the treatment modalities vary. Chemotherapy, radiotherapy, surgery, targeted therapy, and immune therapy are the various treatment for treating oral cancer. Each therapy has its pros and cons. Oral cancer patients undergo various physical and psychological adverse effects which can be short-term or long-term. Cancer survivors face many ill effects like recurrence or secondary tumors along with cardiovascular, renal, and lung complications. The OSCC patients treated with surgery usually have difficulties in swallowing and speech, and also treatment can lead to neuralgia, altered or complete loss of taste sensation. Surgery can also cause cosmetic disfigurement which may require further reconstruction and rehabilitation [3].

Various Indian herbs have been used as anti-cancer agents are Curcuma longa, Zingiber officinale, Cinnamomum verum, Crocus sativus, Ocimum sanctum, Azadirachta indica, and so on. A vital substance present in plants like Cinnamomum cassia (Chinese cinnamon) and Panax ginseng is cinnamic acid, a naturally occurring aromatic carboxylic acid. Cinnamon bark is used to make cinnamic acid. Its structure, which consists of a benzene ring, an alkene double bond, and a functional group of acrylic acid, allows for the modification of the aforementioned functionalities with a wide range of substances to produce bioactive molecules with increased efficacy. It has been discovered that the type of substituents added to cinnamic acid has a significant impact on how biologically effective are synthesized cinnamic acid derivatives [4].

Cinnamic acid possesses anti-cancer, anti-diabetic, anti-inflammatory, anti-bacterial, and neuroprotective effects. By giving the electrons that interact with radicals to produce stable products, cinnamic acid stops the radical chain reactions. Detergents, flavorings, cosmetics, and toiletries all use it as a fragrant element. Enzymatic deamination of phenylalanine can produce cinnamic acid. Derivatives of cinnamic acid have anti-cancer properties and are effective against a variety of malignancies, including breast, colon, and lung cancers. It has been noted that cinnamic compounds have anti-proliferative effects on tumors. Apoptosis is one method used by cinnamic acid derivatives, such as cinnamaldehyde, to kill malignant cells [5].

Through the suppression of the histone deacetylase inhibitor (HDAC enzyme), derivatives of cinnamonyl sulfonamide hydroxamate show selective anti-cancer action in human cancer cells. HDAC inhibitors target microtubules and impair the control of mitotic progression, induce apoptosis, and thus are potent anti-cancer agents. Thus the present study will evaluate the anti-cancer activity of cinnamoyl sulfonamide hydroxamate derivatives against squamous cell carcinoma.

## Materials and methods

The present study is an experimental research, carried out in Santosh Dental College. The material preparation and analysis are carried out in Caritas College of Pharmacy, Kottayam, and the cell culture techniques are carried out in Biogenics, Trivandrum. The study was carried out in various phases. The ethical clearance was obtained with reference 856/PO/Re/S/04/CPSEA.

Design of cinnamoyl hydroxamate derivatives 

(E)-3-(4-(Chlorosulfamoyl) Phenyl) Acrylic Acid Synthesis

Chlorosulfonic acid (0.69g at 5.4 mM) and cinnamic acid (1.0 g, at 0.0068 mM) were mixed at 35 °C for four hours. To keep an eye on the reaction's development, pre-coated thin-layer chromatography (TLC) plates were employed. A beaker containing ice cubes was then filled with the viscous reaction mixture. The resulting yellow precipitate (anhydrous CaCl2) was filtered and it was washed thrice with 20 ml of distilled water and dried in a vacuum to reduce free anhydrous molecules.

(E)-3-(4-(N-(Phenyl Bromo) or (Phenyl Nitro) Sulfamoyl) Phenyl) Acrylic Acid Synthesis

Bromo aniline (0.064g, 4.06 mM) or nitro aniline (0.056, 4.06 mM) were added to a quantity of (E)-3-(4-(chlorosulfamoyl) phenyl) acrylic acid (1 g, 4.06 mM) in 50 ml distilled water, and pH 8 was maintained using aqueous NaHCO3. At 35° C, the reaction mixture was agitated for four hours. The liquid was then adjusted to have a pH of 2 by adding hydrogen chloride (HCl) drop by drop. Product three was recovered as a white precipitate after being washed repeatedly with water, dried, and then ethyl acetate is used to recrystallize.

(E)-N-Hydroxy-3-(4-(N-(Phenyl Bromo) or (Phenyl Nitro) Sulfamoyl) Phenyl) Acrylamide Synthesis

Using a calcium chloride guard tube, compound (E)-N-Hydroxy-3-(4-(N-(phenyl bromo) (1.5 g, 39.26 mM) was added to 30 ml of dichloromethane (Cl2CH2), along with ethyl chloroformate (5.10 g, 47.12 mM) and N-methyl-morpholine (3.9 g, 39.26 mM). The reaction mixture was then agitated at 35 °C for five hours. On a pre-coated TLC plate, the full translation of acrylic acid to acid chloride was seen. Additionally, the addition of freshly made neutral hydroxylamine solution (1.96 g, 58.89 mM) in 30 ml of tetra-hydro-furan (THF) completed the translation of acid chloride to the corresponding hydroxamate derivative. To create the finished product, column chromatography was used to purify the recovered residue, and two compounds were retrieved (3a, 3b) was used for further analysis.

Characterization of synthesized cinnamyl sulfonamide hydroxamate derivatives

Structure Confirmation

Ultra violet visible spectroscopy (UV-Vis) was used to determine the purity of the compound and their absorbance spectrum as they absorb light at specific wavelengths with unique absorbance values. The extracted sample was analyzed using Shimadzu UV-1800 UV spectroscopy. Infrared spectroscopy (IR) was used to identify the molecular structure, identification of chemicals, their chemical bonds, and also their qualitative and quantitative determination of chemicals present. Mass spectroscopy (MS) was done to identify the unknown compounds by molecular weight determination, quantify the known compounds, and also to determine the chemical properties of the molecules present. It was done using Thermo-Fischer scientific mass spectroscopy. For nuclear magnetic resonance (NMR), a thermo-scientific pico spin spectrometer was used to identify the molecules, study the molecular interactions, and also to probe the molecular dynamics (1H and 13C).

Derivatives of synthesized cinnamyl sulfonamide hydroxamate

(E)-N-Hydroxy-3-(4-(N-(phenyl bromo) or (phenyl nitro) sulfamoyl) phenyl) acrylamide synthesis- compound 3a, 3b.

Compound 3a

(E)-N-Hydroxy-3-(4-(N-(phenyl bromo) sulfamoyl) phenyl) acrylamide- percentage yield: 2.37g, 64.56 %; molecular weight: 397g; retardation factor (Rf) value= 0.58; melting point= 184 ± 1 °C; soluble in organic solvents and insoluble in water.

Compound 3b

(E)-N-Hydroxy-3-(4-(N-(phenyl nitro) sulfamoyl) phenyl) acrylamide- percentage yield: 2.64g, 68.93 %; molecular weight: 363g; retardation factor (Rf) value= 0.86; melting point= 144 ± 1 °C; soluble in organic solvents and insoluble in water. (compound 3 was taken as it showed highly positive anti-cancer activity).

To evaluate the in-vitro antioxidant activity of derivatives

Nitric Oxide Scavenging Activity

In the nitric oxide scavenging assay, the sodium nitro prusside is kept in an aqueous solution, and will produce nitric oxide on its own, which will subsequently combine with oxygen to produce nitrite ions, which can be detected using Griess reagent. The reaction between oxygen and nitric oxide scavengers reduces the production of nitrite ions. Spectrophotometry was used to assess the nitric oxide scavenging activity. The reagents used were sodium nitroprusside, Griess reagent, and phosphate-buffered saline (PBS) reagent. Sodium nitroprusside (5 millimol L-1) was mixed with various amounts of samples 3a and 3b in phosphate-buffered saline pH 7.4 before being incubated at 25°C for 30 minutes. Instead of the test drug, distilled water was used as a control in an equivalent amount. After 30 minutes of incubation, 1.5 mL of the incubated solution was removed and diluted with 1.5 mL of Griess reagent (1% sulphanilamide, 2% phosphoric acid, and 0.1% N-1-naphthyl ethylene diamine di-hydro-chloride). By measuring the absorbance of the chromophore produced after the nitrate was diazotized with sulphanilamide and then coupled with N-1 naphthyl ethylene diamine di-hydro-chloride at 546nm, the percentage scavenging activity was calculated in relation to the standard by using % inhibition = control- test/ control x 100.

Reducing Power Assay

The total anti-oxidant effect is calculated using ferric reducing antioxidant assay (FRAP). This method is predicated on the notion that the antioxidant activity grows along with the absorbance of reaction mixtures. The antioxidant compound in the samples is measured at 700 nm by a UV-spectrophotometer with potassium ferricyanide, trichloroacetic acid, and ferric chloride (nitrate with sulphanilamide and subsequent coupling with N-1 naphthyl ethylene diamine di-hydro-chloride was measured at 546 nm, and the percentage scavenging activity was measured with reference to the standard). The reagents used are potassium ferric cyanide, tricarboxylic acid cycle (TCA), ferric chloride, and PBS reagent.

Procedure: Various amounts of samples from concentration of 10mg/mL combined with 2.5ml of phosphate buffer and 2.5ml of 1% potassium ferric cyanide. After that, the mixture was heated at 50°C for 20 minutes. Instead of the test drug, distilled water was used as a control in an equivalent amount. After incubating, 2.5 ml of 10% TCA was added and kept for 10 minutes. After the upper layer (5 ml) was blended with 5 ml of distilled water and 1 ml of 0.1% ferric chloride, the absorbance was measured at 700 nm. Quercetin at 10mg/Ml was used as a reference.

 In-vitro viability, cytotoxicity of synthesized compounds

Viability Analysis

Tryphan blue dye exclusion assay: The tryphan blue dye exclusion test can be used to count the number of live cells present in a cell solution. It is predicated on the notion that live cells will have intact cell walls which block the passage of specific dyes like propidium, Tryphan blue & eosin but dead cells do not. When a dye is merely introduced to a suspension of cells without first visually determining whether the cells are absorbing or rejecting the dye. Viable cells have transparent cytoplasm, while non-viable cells have blue cytoplasm. Preparation of phosphate buffered saline pH 7.4 0.19g of sodium dihydrogen phosphate (NaH2PO4) was mixed with 2.38g 0f disodium hydrogen phosphate (Na2HPO4) and mixed with 8g of sodium chloride and made up to 1000ml with distilled water.

Preparation of cell culture: Human oral cavity squamous cell carcinoma cell line (OECM 1) & a unique head and neck squamous carcinoma cell line (UM- SCC 6) & human oral squamous cell carcinoma forming metastatic foci (HSC-3) cell lines procured from the oral cavity of female Swiss albino mice were used in the study. After 14 days of development, multiplication, and maturation of tumor cells, 5-6 mL of oral mucous fluid was aspirated using an 18G needle and transferred to a centrifuge tube containing PBS and centrifuged for 15 minutes at 800 rpm or at a higher speed for few minutes to avoid increased dead cell count. The pellet was re-suspended in fresh PBS and the process was repeated three times, sufficiently diluted, and used for the study.

Preparation of 0.1% tryphan blue dye: 10 mg tryphan blue dissolved in 100 mL of normal saline. The solution was stored light in an amber color bottle at 40 C. Sample preparation 10 mg of extract was dissolved in 1mL DMSO (sample A). Step I - preparation of higher concentrations of 3a and 3b: 200μg/mL: 780μL of PBS was mixed with 20μL of sample A and 100μL cell line and 100μg/mL: 790μL of PBS was mixed with 10μL of sample A and 100μL cell line. Step II - Preparation of lower concentrations of 3a and 3b: 100μL of sample A was diluted with 900μL of DMSO (sample B) and the following lower concentrations were prepared. 20μg/mL: 780μL of PBS was mixed with 20μL of sample B and 100μL cell line. 10μg/mL: 790μL of PBS was mixed with 10μL of sample B and 100μL cell line. 5μg/mL: 795μL of PBS was mixed with 5μL of sample B and 100μL cell line. Preparation of control 900μL of PBS was mixed with 100μL cell line. The cell suspension was contained in the control tube. They were mixed and incubated for three hours at 370C and 0.1ml of the assay mixture was mixed with 0.1ml of tryphan blue dye and then the percent of non-viable cells was evaluated. The cells were counted using a hemocytometer. The percentage of viability was calculated by dividing the number of viable cells by the total number of cells and multiple by 100.

Analysis of cytotoxicity

MTT Assay

The MTT assay was carried out using OECM 1, UM-SCC 6 & HSC 3 cell lines. The various reagents used are MTT reagent, dimethyl sulfoxide (DMSO), and Dulbecco's modified eagle medium (DMEM). The OECM 1 and UM- SCC 6 and HSC 3 cells were cultured in DMEM. Cell lines were grown in a 25 cm2 tissue culture flask with an antibiotic solution containing amphotericin, penicillin, and streptomycin as well as DMEM supplemented with 10% FBS, L-glutamine, and sodium bicarbonate. Cultured cell lines were maintained at 37°C in an incubator with humidified 5% CO2. Through direct observation of the cells using an inverted phase contrast microscope and the MTT assay method, the vitality of the cells was assessed.

Materials required: OECM 1, UM- SCC 6 & HSC 3 Cell lines, MTT reagent, DMSO, DMEM medium.

Procedure: Seeding of cells in a 96-well plate trypsinized two-day-old confluent monolayer of cells was sown in 96-well tissue culture plates at a density of 5 x 104 cells per well. The cells were then incubated at 37 °C on a humidified 5% CO2 incubator.

Compound stock preparation: Using a cyclomixer, 1mg of each of the test substances 3a and 3b were weighed and dissolved in 1mL DMEM. To guarantee sterility, a 0.22 m Millipore syringe filter was used to filter the sample solution. After 24 hours, the growth media was withdrawn for the anticancer evaluation. Five freshly produced compounds in 5% DMEM were serially diluted two folds five times (100g, 50g, 25g, 12.5g, and 6.25g in 500l of 5% DMEM). After receiving treatment for 24 hours, the complete plate was examined under an inverted phase contrast tissue culture microscope for an anticancer assay, and microscopic observations were captured as photographs. Cellular morphological alterations like rounding or shrinkage, granulation, and vacuolization in the cytoplasm were all taken into account as indications of cytotoxicity.

## Results

Statistical analysis was performed using SSPSS (IBM Corp. Released 2013. IBM SPSS Statistics for Windows, Version 22.0. Armonk, NY: IBM Corp). A chi-square test was carried out to check for any statistical difference between the observed value and the expected value among the control and study group. One-way ANOVA analysis was carried out to compare the means and determine the association between the means of the study group. Post-hoc Tukey HSD (beta) was performed to facilitate pairwise comparisons within the analysis of variance (ANOVA) data. The level of significance [P-Value] was set at P<0.05.

Cinnamyl sulfonamide hydroxamate derivatives

Compound 3a

(E)-N-Hydroxy-3-(4-(N-(phenyl bromo) sulfamoyl) phenyl) acrylamide- percentage yield: 2.37g, 64.56 %; molecular weight: 397g; retardation factor (Rf) value= 0.58; melting point= 184 ± 1 °C; soluble in organic solvents and insoluble in water.

Compound 3b

(E)-N-Hydroxy-3-(4-(N-(phenyl nitro) sulfamoyl) phenyl) acrylamide- percentage yield: 2.64g, 68.93 %; molecular weight: 363g; retardation factor (Rf) value= 0.86; melting point= 144 ± 1 °C; soluble in organic solvents and insoluble in water.

Structural characterization of synthesized cinnamyl sulfonamide hydroxamate derivatives

The C=O stretching was present at 1672, the C-S group was present at 672 cm-1, and the presence of the O=S=O group was indicated by a peak at 1158 cm-1 in the FTIR spectra of the 3a. O-H and N-H groups are present, as shown by the peaks at 3260 and 2676. A peak at 3360 cm-1 and another at 3327 cm-1 in compound 3b's FTIR spectrum suggest the existence of O-H and N-H groups, respectively. Peaks for C=O at 169 cm-1 and for C-S at 697 cm-1 are also produced by the compound. The presence of the S=O group is indicated by the peak at 1179 cm-1. The synthetic compounds 3a and 3b's 1H NMR spectra reveal the existence of 10 aromatic protons in the 6-8 ppm region. Both compounds had a peak for N-H protons at 10 ppm. The synthetic compounds 3a and 3b's 13C NMR spectra reveal the existence of C=O at a peak value of 167.50 and 168.11, respectively. The ranges of 124.44-142.37 and 14.36-129.12, respectively, contain the peaks in 3a and 3b showing the presence of aromatic carbons, respectively. The molecular ion peak may be seen as the base peak at m/z 398 and 365 in the mass spectra of compounds 3a and 3b. Several spectroscopic analytical techniques were used to confirm the structure of the synthesized molecules.

Nitric oxide scavenging assay

Anti-oxidant activity can be analyzed by determining the nitric oxide radical inhibition concentration carried out by 3a and 3b (two synthetic derivatives) with gallic acid as standard at reductive potential concentration (Table 1). The dose-dependent activity and % of inhibition are given as follows:

**Table 1:** Nitric oxide radical inhibition concentration of standard gallic acid

Group	Concentration (µg/mL)	Absorbance (Mean± SD)	% Inhibition/ Conc
Standard Control Gallic Acid	6.25	0.24±0.005	17.5
	12.5	0.19±0.004	34.9
	25	0.16±0.002	48.54
	50	0.08±0.002	67.08
	100	0.04±0.001	74.53
	200	0.02±0.001	95.7
IC_50_	29.27 µg/Ml		

The percentage inhibition of the standard gallic acid increased with increasing concentration of the compound assay and showed maximum inhibition of 95.7% at 200 μg/mL concentration and a minimum of 17.5% at 6.25μg/mL concentrations. Correspondingly, for both test compounds 3a and 3b, the inhibition (%) percentage scavenging increases linearly with concentration at the given cell phase. The half-maximal inhibitory concentration (IC50 value for the standard gallic acid was found to be 29.27μg/mL whereas the IC50 values of test compounds 3a and 3b were found to be 81.27μg/mL and 129.27μg/mL respectively among which 3a was found to be good antioxidant than 3b (Tables 2, 3). 

**Table 2:** Nitric oxide radical inhibition concentration of compound 3a

Group	Concentration (µg/mL)	Absorbance (Mean± SD)	% Inhibition/ Conc
Compound 3a	6.25	0.54±0.004	12.5
	12.5	0.46±0.003	26.6
	25	0.39±0.006	31.4
	50	0.25±0.003	45.41
	100	0.18±0.001	63.72
	200	0.12±0.001	79.46
IC_50_	81.27 µg/Ml		

**Table 3:** Nitric oxide radical inhibition concentration of compound 3b

Group	Concentration (µg/mL)	Absorbance (Mean± SD)	% Inhibition/ Conc
Compound 3b	6.25	0.51±0.004	9.5
	12.5	0.40±0.003	18.4
	25	0.32±0.006	26.74
	50	0.27±0.003	33.91
	100	0.19±0.001	46.11
	200	0.14±0.001	69.38
IC_50_	129.27 µg/Ml		

Reducing power assay using standard quercetin test compound

Reducing potential was assessed by absorbance of different concentrations of compounds 3a and 3b at the given environment under standard Quercetin compound measured at 700nm (Table 4).

**Table 4:** Reducing power assay of the standard (quercetin)

Group	Concentration (µg/mL)	Absorbance (Mean± SD)
Standard Control Quercetin	6.25	0.95±0.003
	12.5	1.19±0.002
	25	1.76±0.002
	50	2.18±0.005
	100	2.64±0.006
	200	2.82±0.005

The test compound 3a and 3b (Tables 5, 6) shows maximum absorbance (mean± SD) of 2.38 and 2.12 ± 0.001 and 0.006 respectively at 200μg/mL and the standard control using quercetin shows maximum absorbance at the concentration of 200μg/mL (mean± SD) about 2.82 ± 0.005. This signifies that the reducing power of compound 3a was comparatively higher than that of 3b with standard control as quercetin. 

**Table 5:** Reducing power assay of the compound 3a

Group	Concentration (µg/mL)	Absorbance (Mean± SD)
Compound 3a	6.25	0.47±0.005
	12.5	0.98±0.004
	25	1.44±0.002
	50	1.86±0.003
	100	2.07±0.006
	200	2.38±0.001

**Table 6:** Reducing power assay of the compound 3b

Group	Concentration (µg/mL)	Absorbance (Mean± SD)
Compound 3b	6.25	0.42±0.001
	12.5	0.81±0.002
	25	1.27±0.002
	50	1.56±0.004
	100	2.04±0.003
	200	2.12±0.006

Tryphan blue

The tryphan Blue dye exclusion test can be used to count the number of sentient cells present in a given cell line suspension or solution. The OECM 1 & UM- SCC 6 & HSC-3 cell lines were cultured as per standard procedures described earlier and stained using tryphan blue to check the viability when treated with compounds 3a and 3b. In this method, dead cells appear dark blue as they take up the stain color. This stain allows the differentiation of dead cells and cells with damaged cell membranes with viable or live cells that do not take up the stain (unstained). This examines the cell suspension depending on the integrity of the cellular membrane exposed to compounds 3a and 3b in the present study with the percentage viability expressed as mean± SD (Tables 7, 8, 9).

**Table 7:** OECM-1 cell line- percentage inhibition of cell viability by compounds 3a and 3b OECM 1: human oral cavity squamous cell carcinoma cell line

Concentration (μg/mL)	% Inhibition of cell viability of compounds	
	3a	3b
6.25	12.6	14.52
12.5	19.2	27.86
25	24.93	33.91
50	41.89	38.84
100	65.78	42.58
200	73.45	63.19
LC_50_	79.637 µg/mL	160.051 µg/Ml

**Table 8:** UM-SCC 6 cell line- percentage inhibition of cell viability by compounds 3a and 3b UM SCC 6:  a unique head and neck squamous carcinoma cell line

Concentration (μg/mL)	% Inhibition of cell viability of compounds	
	3a	3b
6.25	11.2	18.34
12.5	19.56	23.48
25	25.4	29.87
50	39.87	34.79
100	58.36	41.63
200	66.57	62.49
LC_50_	94.235 µg/mL	179.152 µg/mL

**Table 9:** HSC- 3 cell line- percentage inhibition of cell viability by compounds 3a and 3b *Highly cytotoxic; HSC 3: Human oral squamous cell carcinoma forming metastatic foci

Concentration (μg/mL)	% Inhibition of cell viability of compounds	
	3a	3b
6.25	14.35	15.6
12.5	25.65	21.3
25	38.43	24.27
50	53.26	39.7
100	69.17	55.48
200	76.31	63.28
LC_50_	43.519 µg/mL*	92.391 µg/mL

The test compounds 3a and 3b showed a concentration-dependent decrease in percentage cell viability with an increase in concentration in OECM 1 & UM- SCC 6 & HSC-3 cell lines. The test compound 3b produces maximum percentage inhibition of viability 63.19%, 62.49%, and 63.28% at 200μg/mL respectively, and minimum inhibition of cell viability 14.52%, 18.34%, and 15.6% at 6.25μg/mL concentration. Whereas 3a showed a maximum percentage inhibition of cell viability of 73.45%, 66.57%, and 76.31% at 200μg/mL and a minimum percentage inhibition of cell viability of 12.6%, 11.2%, and 14.35% at 6.5μg/ml (Tables 7, 8, 9).

The two test compounds 3a and 3b caused significant cytotoxicity in OECM 1 & UM- SCC 6 & HSC-3 cell lines incubated with phosphate-buffered saline. The lethal concentration (LC) 50 values were found to be 79.637 µg/mL (3a) and 160.051 µg/mL (3b) for OECM-1 followed by 94.235 µg/mL (3a) and 179.152 µg/mL (3b) for UM-SCC 6 with higher toxicity at HSC-3 cell line that was found to be 43.519 µg/mL (3a) and 92.391 µg/mL (3b) lethal even at a very low concentration. Among these, the test compounds, 3a produces more cytotoxicity towards the HSC-3 cell line followed by the OECM-1 cell line and the least being 3b compound at the UM-SCC6 cell line (160.051 μg/ml at 200 μg/ml concentration).

MTT((3-[4,5-dimethylthiazol-2-yl]-2,5 diphenyl tetrazolium bromide) assay

MTT assay was carried out to evaluate the inhibition concentration and measure the viable cells by determining the mitochondrial activity after 24 hours of specified compound concentration treatment with MTT assay expressed as mean ± SD (Tables 10, 11, 12).

**Table 10:** Percentage inhibition of 3a and 3b on growth of OECM 1 Cell line OECM 1: human oral cavity squamous cell carcinoma cell line

Groups	Concentration (µg/Ml)	Absorbance (Mean ± SD)	Percentage Inhibition (%)
3a	6.25	2.78±0.004	94.5
	12.5	2.12±0.012	84.63
	25	1.98±0.008	71.56
	50	1.23±0.002	58.9
	100	0.87±0.013	41.83
	200	0.47±0.005	24.68
IC_50_	78.562 µg/mL		
3b	6.25	2.09±0.004	90.4
	12.5	1.93±0.012	82.36
	25	1.78±0.008	79.47
	50	1.14±0.002	76.12
	100	0.93±0.013	63.54
	200	0.42±0.005	47.33
IC_50_	129.361 µg/mL		

**Table 11:** Percentage inhibition of 3a and 3b on growth of UM- SCC 6 Cell line UM SCC 6:  a unique head and neck squamous carcinoma cell line

Groups	Concentration (µg/Ml)	Absorbance (Mean ± SD)	Percentage Inhibition (%)
3a	6.25	1.98±0.012	92.71
	12.5	1.72±0.008	85.4
	25	1.55±0.007	72.38
	50	1.29±0.011	63.89
	100	0.87±0.006	46.73
	200	0.43±0.009	39.56
IC_50_	79.335 µg/mL		
3b	6.25	2.12±0.007	91.9
	12.5	1.93±0.006	88.56
	25	1.67±0.012	82.37
	50	1.35±0.006	78.54
	100	0.93±0.007	59.71
	200	0.78±0.005	44.98
IC_50_	157.685 µg/mLTable 11: Percentage inhibition of 3a and 3b on growth of UM- SCC 6 Cell line		

**Table 12:** Percentage inhibition of 3a and 3b on growth of HSC-3 Cell line HSC 3: Human oral squamous cell carcinoma forming metastatic foci

Groups	Concentration (µg/Ml)	Absorbance (Mean ± SD)	Percentage Inhibition (%)
3a	6.25	2.78±0.004	90.23
	12.5	2.12±0.012	79.16
	25	1.98±0.008	67.12
	50	1.23±0.002	48.63
	100	0.87±0.013	41.83
	200	0.47±0.005	24.68
IC_50_	46.802 µg/mL		
3b	6.25	2.78±0.004	89.26
	12.5	2.12±0.012	81.48
	25	1.98±0.008	72.6
	50	1.23±0.002	63.89
	100	0.87±0.013	59.17
	200	0.47±0.005	35.86
IC_50_	108.521 µg/mL		

## Discussion

Evidence-based studies showed cinnamic acid novel targeted derivatives exhibit anti-cancerous potential through HDAC inhibition by selective and less toxic molecular mechanisms. In the present study, two cinnamyl sulfonamide hydroxamate compounds (derivatives 3a, 3b) were assessed to explore the potential anti-cancerous activity of cinnamic acid derivatives. Pontiki et al. [6] observed potential anti-cancerous activity by novel synthesis method and structural configuration of cinnamic acid derivatives while a study by Reddy et al. showed 3 cinnamyl sulfonamide hydroxamate (CSH) products induces HDAC inhibition and possible activation of apoptotic pathways thus providing anti-cancerous and anti-inflammatory activity [7].

A nitrous oxide scavenging assay was performed to evaluate the percentage inhibition of the antioxidant and free radical scavenging activity. Sarwar et al. observed good antioxidant activity with IC50 of 55.4 ± 0.21 μg/mL among Quercus incana roxb that increased with increasing concentration of the compound assay. Gryko et al. [8] in cinnamic acid derivatives (tHCA, dHCA) and natural cinnamic acid observed 2,2-diphenyl-1-picrylhydrazyl (DPPH), hydroxyl (HO) radicals scavenging activity and Fe3+ reduction ability at 10Mm (4-HCA), 0.05Mm (3, 4, 5-tHCA,dHCA) showing IC50 values of 49.99 ± 0.58, 50.22 ± 0.55 and 53.02 ± 0.80 with 80% of initial concentration at 100 μg/mL. In the present study, maximum inhibition of 95.7% occurred at 200 μg/mL concentration using standard gallic acid whereas the IC50 values of cinnamic derivative compounds 3a (OH derivatives) and 3b (-OH-OH derivatives) were found to be 81.27μg/mL and 129.27μg/mL respectively. Thus signifying derivatives of cinnamic acid as a potential scavenger with antiradical activity enhanced by an increased number of hydroxyl derivative groups (-OH p-hydroxy < dihydroxy < hydroxydimethyl) caused by a decrease in the kinetic stability, and influence of aromatic substitution as a result of symmetrization of the electronic charge dissemination of these 3a and 3b compound molecules [9].

Reducing potential was assessed by absorbance of different concentrations of compounds 3a and 3b at the given environment showed maximum absorbance (mean± SD) of 2.38 and 2.12 ± 0.001 and 0.006 respectively at 200μg/mL. Barre et al. in a study to evaluate the reduction potential and polypharmacy of cinnamic acid derivatives in type 2 diabetes mellitus (DM) patients observed derivatives such as chlorogenic acid, cinnamaldehyde, caffeic acid, and ferulic acid exhibited maximum reduction at lower concentrations [10]. Adisakwattana in a similar study on diabetic individuals observed the reduction potential of p-methoxy cinnamic acid at 10-100 μM concentration (11.5%) and caffeic acid (50.1%) at a slightly higher concentration [11]. Pellerito et al. showed a marked reduction at 400 nM even up to 1 µM concentration. These results suggest that the replacement of aromatic (substitute) by hydroxyl group traps the dicarbonyl’s thus enhancing the reduction potential even at lower concentrations [12]. Our results also support the findings that biochemical reactions of specific compounds play an important role in the up-regulation of apoptotic pathways and also the regulation of terminal differentiation pathways.

A tryphan blue dye exclusion test was carried out to assess the number of sentinel cells present in the given OECM 1 & UM- SCC 6 & HSC-3 cell line suspension. Rodrigo et al. in a study employed cell lines derived for human oral SCC’s to assess the effect of raspberry-ethanol extract (RO-ET) activity on cellular characteristics using tryphan blue exclusion dye test observed cell proliferation without disorientation in the viability and cell suspension inhibition at 10, 50, and 100 μg/ml (> 97%) concentration that induces both apoptosis and differentiation [13]. Ladke et al., Chan et al. recommended the use of tryphan blue for effective preservation of cell line integrity from contamination, retaining the tissue architecture, staining dead cells, viability inhibition potential, and microenvironment characterization of cell therapy. The present study showed maximum percentage inhibition of viability at 63.19%, 62.49%, and 63.28% at 200μg/mL respectively among the cinnamic derivative compounds [14, 15]. This could be attributed to concentrate gradient differentiation and membrane-compromised dead cells in the cell lines used in the present study nonetheless acceptable level of viability was observed where the appearance of live cells was not affected comparable to morphological changes for typical cancer cell cultures at high sustainability.

MTT assay was carried out to evaluate the inhibition concentration and measure the viable cells by determining the mitochondrial activity after 24 hours of specified compound concentration treatment with MTT assay expressed as mean ± SD. In a study by Jayashree et al. 2-Quinolone Schiff's bases in a novel series of constituents (15 chemicals) were produced. By using the MTT assay method, the active compounds were examined for their antiproliferative ability against lung cancer cell lines [16]. The bromo and bromo aniline derivatives were shown to be more cytotoxic among the 15 synthetic chemicals studied. Kaur et al. designed and synthesized 16 novel cinnamic acid derivatives and evaluated them for in vitro cytotoxicity (lung cancer cell line, A-549 cell culture line) using MTT assay [17]. The study showed substantial docking interactions, selective MMP-9 inhibitors as potential antineoplastic agents, and also by binding patterns with MMP-9 protein suggesting the in vitro cytotoxicity outcome nature. Mingoia et al. [18] analyzed the potential wound healing property of novel cinnamic acid derivatives using MTT assay on keratinocytes with the study compounds and revealed MTT viability assay as an effective test in evaluating the effects of derivatives in proliferation and viability at specific concentrations. Cheng et al. [19] tested the inhibitory growth potential of OECM-1 and SAS Cell lines treated with prodigiosin (PG) using MTT assay and reported a significant level of cytotoxicity with 0.4 μM of PG and 3 methyladenine (1 and 5 mM) in OECM-1 while Ardito et al. detected reduction in proliferation potential at increasing concentration and duration (24hrs, 48hrs, and 72 hrs) respectively with increasing concentration of genistein in HSC-3 cell line with an IC50 of 22µM [20].

In the present study, LC 50 values of compound (OECM 1: 3a: 79.637 µg/mL; 3b: 160.051 µg/mL) (UM-SCC 1: 3a: 94.235 µg/mL; 3b: 179.152 µg/mL) and (HSC- 3: 3a: 43.519 µg/mL; 3b: 92.391 µg/mL) for 24 hours were observed respectively thus suggesting a higher metabolic activity of compound 3a on the cell lines as an indicator of cell viability, proliferation, and cytotoxicity compared to compound 3b and high resistance potential displayed by these cell lines in response to the compounds 3a and 3b. Varadarajan et al. performed an in-vitro study to assess the anticancer property of cinnamic acid extracts using MTT assay (3-(4,5-dimethylthiazol-2-yl)-2,5-diphenyltetrazolium bromide) and observed anti-cancerous effect of 4-hydroxy-cinnamic acid and cinnamaldehyde through apoptosis by specific mitochondrial alteration in membrane potential and s-phase arrest in cell cycle [21].

Reddy et al. in an assessment of synthesized cinnamyl derivatives (NMJ-1, -2, and -3) study showed cancer cell cytotoxicity with an effective increase in the apoptotic index and arrest in the G2/M phase in the cell cycle. Induction of p21 (Waf1/Cip1) expression, hyper-acetylation of H3 histidione, inhibition of induced MMP-2, and 9 expressions. In accordance with these results the present study also on the control treatment of compounds 3a and 3b with anisidine and toluidine revealed a statistically significant difference at G0/G1 phase and S phase. A significant decrease in cells in G0/G1 phase from 70.4% to 34.0 (%Gated) and an increase in cells in the S phase from 14.8% to 38.5% (gated %) was observed suggesting S phase arrest [22].

Sova et al. demonstrated various biological effects of cinnamic acid derivatives (CAD) and cytotoxicity on HeLa, K562, Fem-x, and MCF-7 cell lines using MTT assay and showed significant cytotoxicity of the CAD compounds on the malignant cell lines [23]. Cell study analysis using flow Cytometry revealed an accumulation of cells in the G0/G1 phase followed by disruption of the cell cycle phase of viable cells in the given cell line. Similarly in the present study a marginal decrease in cells in the G0/G1 phase from 70.4% to 53.1 (%Gated) and an increase in cells in the S phase from 14.8% to 18.0% (Gated %) and at G2 M phase cells (10.9% to 14.5%) indicative of both late apoptotic and necrotic cell population when compared with untreated control cells suggesting a marginal arrest comparatively lower than anisidine at S phase.

Plants are having effective medicinal value with reduced adverse effects. Future studies on the efficacy of the drug, mode of drug release and targeted drug delivery can be carried out using animal models. The extracted study molecule can also be compared with commercial anti-cancer agents for an effective drug delivery system. 

## Conclusions

Exploring the targeted therapy and less harmful chemicals to treat cancer is a never-ending task. Recent rapid scientific advancements have improved our knowledge of cancer biology. As a result, a number of evolutionary targets have been found. The discovery of histone deacetylase as a new and potential target for cancer treatment. By inhibiting HDAC, cinnamic acid compounds demonstrate their anticancer effectiveness. Novel cinnamyl sulfonamide hydroxamate derivatives 3a and 3b were synthesized, characterized, and screened for their in vitro and in vivo anticancer potential. The compounds were subjected to anti-oxidant assays like ferric reducing power assay and NO scavenging assay. The test compounds 3a and 3b showed a concentration-dependent increase in the absorbance of Fe2+ in reducing power assay. MTT assay was carried out to evaluate the inhibition concentration and measure the viable cells, suggesting a higher metabolic activity of compound 3a on the cell lines as an indicator of cell viability, proliferation, and cytotoxicity compared to compound 3b and high resistance potential displayed by these cell lines in response to the compounds 3a and 3b. 
